# Altered 5-HT_2A/C_ receptor binding in the medulla oblongata in the sudden infant death syndrome (SIDS): Part II. Age-associated alterations in serotonin receptor binding profiles within medullary nuclei supporting cardiorespiratory homeostasis

**DOI:** 10.1093/jnen/nlae004

**Published:** 2024-02-07

**Authors:** Kevin J Cummings, James C Leiter, Felicia L Trachtenberg, Benjamin W Okaty, Robert A Darnall, Elisabeth A Haas, Ronald M Harper, Eugene E Nattie, Henry F Krous, Othon J Mena, George B Richerson, Susan M Dymecki, Hannah C Kinney, Robin L Haynes

**Affiliations:** Department of Biomedical Sciences, Dalton Cardiovascular Research Center, University of Missouri, Columbia, Missouri, USA; Department of Molecular and Systems Biology, Geisel School of Medicine at Dartmouth, Hanover, New Hampshire, USA; Clinical Research, Carelon Research, Newton, Massachusetts, USA; Department of Genetics, Harvard Medical School, Boston, Massachusetts, USA; Department of Molecular and Systems Biology, Geisel School of Medicine at Dartmouth, Hanover, New Hampshire, USA; Department of Research, Rady’s Children’s Hospital, San Diego, California, USA; Department of Neurobiology and the Brain Research Institute, David Geffen School of Medicine at UCLA, Los Angeles, California, USA; Department of Molecular and Systems Biology, Geisel School of Medicine at Dartmouth, Hanover, New Hampshire, USA; Department of Pediatrics, University of California San Diego, San Diego, California, USA; Departments of Pathology and Pediatrics, Rady Children’s Hospital, San Diego, California, USA; San Diego County Medical Examiner Office, San Diego, California, USA; Departments of Neurology and Molecular Physiology & Biophysics, University of Iowa, Iowa City, Iowa, USA; Department of Genetics, Harvard Medical School, Boston, Massachusetts, USA; Department of Pathology, CJ Murphy Laboratory for SIDS Research, Boston Children’s Hospital and Harvard Medical School, Boston, Massachusetts, USA; Department of Pathology, CJ Murphy Laboratory for SIDS Research, Boston Children’s Hospital and Harvard Medical School, Boston, Massachusetts, USA

**Keywords:** Autoradiography, Blood pressure recovery, Gasping, Hypoxia, Inferior olive, Nucleus of the solitary tract, Raphe

## Abstract

The failure of chemoreflexes, arousal, and/or autoresuscitation to asphyxia may underlie some sudden infant death syndrome (SIDS) cases. In Part I, we showed that some SIDS infants had altered 5-hydroxytryptamine (5-HT)_2A/C_ receptor binding in medullary nuclei supporting chemoreflexes, arousal, and autoresuscitation. Here, using the same dataset, we tested the hypotheses that the prevalence of low 5-HT_1A_ and/or 5-HT_2A/C_ receptor binding (defined as levels below the 95% confidence interval of controls—a new approach), and the percentages of nuclei affected are greater in SIDS versus controls, and that the distribution of low binding varied with age of death. The prevalence and percentage of nuclei with low 5-HT_1A_ and 5-HT_2A/C_ binding in SIDS were twice that of controls. The percentage of nuclei with low 5-HT_2A/C_ binding was greater in older SIDS infants. In >80% of older SIDS infants, low 5-HT_2A/C_ binding characterized the hypoglossal nucleus, vagal dorsal nucleus, nucleus of solitary tract, and nuclei of the olivocerebellar subnetwork (important for blood pressure regulation). Together, our findings from SIDS infants and from animal models of serotonergic dysfunction suggest that some SIDS cases represent a serotonopathy. We present new hypotheses, yet to be tested, about how defects within serotonergic subnetworks may lead to SIDS.

## INTRODUCTION

The sudden infant death syndrome (SIDS) is characterized by the sudden, unexpected death of an infant in the first postnatal year that remains unexplained by complete autopsy and forensic investigation (1). SIDS infants, who are seemingly normal, are found dead unexpectedly, typically during a sleep period (2). SIDS remains the leading cause of post-neonatal infant mortality in the United States (3). Over the last 2 decades, the rate of SIDS has not declined despite increased public health campaigns promoting awareness of safe infant sleep (e.g. supine position) and infant care practices that may reduce the risk of SIDS (e.g. breastfeeding (4, 5)). Instead, the overall SIDS rate has plateaued in the United States (6) and has even increased recently in African-American infants (7). There is an urgent need to establish the definitive cause(s) and basic mechanism(s) of SIDS, upon which specific therapeutic remedies and more effective means of prevention can be developed (6).

During sleep, infants and adults alike can be subjected to intermittent blood gas disturbances due to re-breathing (i.e. breathing in exhaled air), a brief loss of airway patency (i.e. obstructive sleep apnea), or events in the central nervous system that halt the activity of the diaphragm (i.e. central apnea). The first line of defense is activation of chemoreceptors triggered by hypoxia and elevated tissue carbon dioxide (CO_2_) with associated acidosis. Chemoreceptor activation provides excitatory signals to regions of the brain that promote arousal from sleep (8–10). The activation of chemoreceptors also increases neural activity within respiratory and cardiovascular networks that together combat the blood gas disturbance (11–14). If this first line of defense fails or is insufficient, hypercapnia and hypoxia (i.e. asphyxia) become progressively more severe, potentially leading to hypoxic coma (15). Survival then hinges on a complex behavior (autoresuscitation) relying on multiple, integrated physiological processes to support reoxygenation of the brain to allow the restoration of normal breathing (eupnea) and ultimately reversal of hypoxic coma.

There is a wealth of pathological and molecular evidence that SIDS infants experience chronic hypoxia, perhaps linked to repeated periods of re-breathing, or obstructive or central apnea and bradycardia, prior to a terminal event. Brainstem gliosis (16–18) and elevated hypoxic markers have been identified in a variety of tissues, suggesting that SIDS infants were chronically hypoxic prior to the terminal event (19–23). Analyses of cardiorespiratory records obtained immediately prior to death suggest that the final event often involved acute, severe asphyxia that the infant could not overcome. For example, prolonged apnea, bradycardia, and gasping (all indicating severe asphyxia) have been observed in SIDS infants prior to death (24, 25). In a normal infant, the integrated physiological components of arousal and autoresuscitation are successful; eupnea and consciousness are restored. In at least some SIDS cases, after hypoxia and/or CO_2_-induced arousal failed or was ineffective, gasping was not sufficient to restore cardiorespiratory function, leading to death.

Neuropathological evidence obtained from SIDS infants by our group over the last 3 decades is in keeping with the concept that SIDS is associated with decreased signaling from serotonin (5-hydroxytryptamine [5-HT]), a monoamine neuromodulator, in key medullary nuclei supporting arousal and autoresuscitation. Neurons that synthesize 5-HT reside in the medulla, pons, and midbrain and regulate diverse functions within the CNS. We initially reported reduced 5-HT receptor binding using ^3^H-lysergic acid diethylamide, a relatively nonspecific 5-HT receptor ligand (26, 27). SIDS infants have reduced 5-HT in the raphe obscurus (ROb) and the nucleus paragigantocellularis lateralis (PGCL), two medullary 5-HT source nuclei (i.e. those nuclei containing serotonergic neuronal cell bodies) that release 5-HT through projections to target nuclei (i.e. those nuclei containing 5-HT receptors but not serotonergic cell bodies) (28). Moreover, tryptophan hydroxylase 2 (TPH2), the enzyme responsible for the bulk of 5-HT synthesis in the central nervous system, is reduced in the ROb of SIDS infants compared to controls (28). Various isoforms of 14-3-3, a family of regulatory proteins with multiple functions, including regulating the activity of TPH2, are also reduced in the nucleus gigantocellularis (GC) of SIDS infants (29).

Based on the findings from SIDS infants, we have more recently used animal models to investigate hypotheses positing that serotonergic defects in key neuromodulatory systems regulating CO_2_ chemoreception, arousal (the first line of defense), and autoresuscitation (the last resort) underlie some cases of SIDS. As a group, we have shown that animal models of serotonergic dysfunction display defects in CO_2_ chemoreception (14, 30–33), arousal (8, 34–36), and autoresuscitation, with animals unable to recover heart rate, blood pressure, eupnea, and consciousness following multiple episodes of asphyxia (37–41). These findings support the concept that abnormal 5-HT receptor binding in SIDS reflects serotonergic dysfunction in these key medullary nuclei, compromising these vital processes that normally protect an infant during sleep periods.

The current paper is the second of a 2-paper series. In Part I, we presented data demonstrating that 5-HT_2A/C_ receptor binding was reduced in nuclei comprising tegmental and olivocerebellar subnetworks of SIDS infants compared to age-adjusted autopsy controls; both subnetworks are 5-HT targets and participate in arousal and cardiorespiratory reflexes. For some nuclei, reduced receptor binding was dependent on age, with the greatest reduction at the oldest ages. Over the course of our research, we came to appreciate that the affected medullary pathways in the SIDS cases share two critical features: (1) They are all involved in protective responses that ultimately help to restore oxygen (O_2_) and CO_2_ status of vital tissues (e.g. brain) during a cardiorespiratory event, including during sleep (e.g. apnea); and (2) 5-HT and the neurons that produce it play an essential role in these processes (30–33, 36, 38, 39, 41–46). Based on the findings described in Part I, we proposed the existence of an integrative brainstem network that in SIDS infants fails to preserve breathing, facilitate arousal, and/or induce successful autoresuscitation. In Part II of this series, we address the overall hypothesis that 5-HT_1A_ and 5-HT_2A/C_ receptor binding, statistically defined as low, manifest differently in SIDS in key source and target nuclei, in a manner depending on age at death. Here, in a continued analysis of the Part I database, we address, for the first time, the prevalence of low 5-HT receptor binding in SIDS infants across nuclei and within different age bins. We more deeply examine our autoradiography data from Part I, given the unique features of this precious SIDS autopsy cohort, the likes of which is becoming progressively more difficult to obtain due to decreasing autopsy rates and general complications in obtaining parental consent during the period between death and autopsy.

## MATERIALS AND METHODS

### Clinicopathologic database and tissue processing for receptor autoradiography

**Figure 1.**
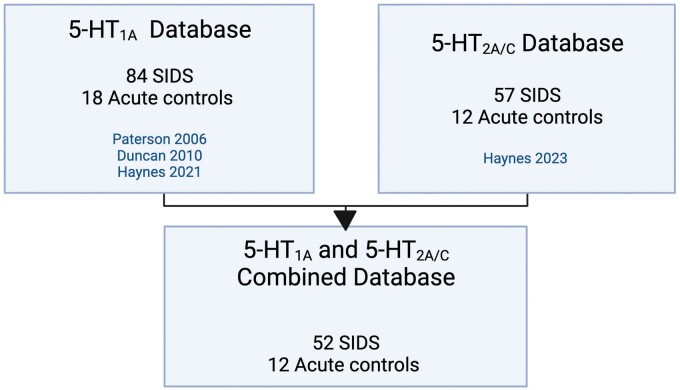
The diagram illustrates the different laboratory databases utilized in this study and the numbers of SIDS and control cases within each database. The individual databases are comprised of infants with 5-HT_1A_ or 5-HT_2A/C_ binding data from receptor ligand autoradiography. The combined database is comprised of infants that have both 5-HT_1A_ and 5-HT_2A/C_ binding data. Created with Biorender.com.

Historically, we accrued autopsy samples continuously and created independent datasets when enough were present to merit analysis. The published 5-HT_1A_ and 5-HT_2A/C_ data were obtained from cohorts of SIDS infants and controls previously collected in our lab over 3 different time periods and designated as independent datasets (datasets 3–5 from our laboratory). The 5-HT_1A_ receptor binding database used here comprised data from SIDS and controls from datasets 3, 4, and 5 and the 5-HT_2A/C_ receptor binding database used here comprised SIDS and controls from datasets 4 and 5. As depicted in Figure 1, a combined database was derived from infants in the foregoing individual databases who had both 5-HT_1A_ and 5-HT_2A/C_-binding receptor measurements.

**Figure 2.**
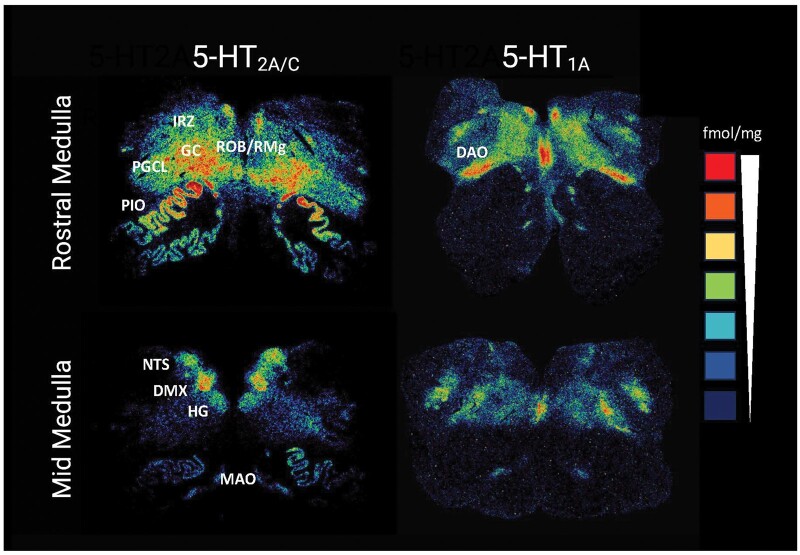
Representative autoradiograms of ^125^I-DOI binding to 5-HT_2A/C_ receptors and ^3^H-DPAT binding to 5-HT_1A_ receptors in a 53 postconceptional week SIDS infant. Mid and rostral levels of the medulla are shown and measured nuclei are labeled. A representation of a radioactivity standard is shown with fentamol/mg (fmol/mg) given from high binding (red) to low binding (dark blue). Binding to 5-HT_2A/C_ receptors is heavily concentrated in the reticular formation, that is IRZ, PGCL, and GC, in the rostral medulla compared to low binding in the reticular formation of the mid-medulla. This finding is relevant to the chemoarchitecture of gasping because the 5-HT_2A_ receptor is essential for gasping. HG, hypoglossal nucleus; NTS, nucleus of the solitary tract; DMX, dorsal motor nucleus of vagus; PIO, principal inferior olive; MAO, medial accessory olive; DAO, dorsal accessory olive; RO, raphe obscurus; GC, nucleus gigantocellularis; IRZ, intermediate reticular zone; PGCL, nucleus paragigantocellularis lateralis. The figure is reproduced from Haynes et al (47) with modifications.

All brainstems previously analyzed came from the Office of the Chief Medical Examiner, San Diego, CA and were available for research under the auspice of the California Code, Section 27491.41. The reader is referred to Part I for the definition and adjudication of SIDS infants and autopsy controls, the protocol for tissue processing and sectioning, methods for 5-HT_1A_ and 5HT_2A/C_ receptor autoradiography, choice of atlases for human brainstem anatomy, and tabulation of nuclei in the medulla that were sampled for receptor binding levels (47). Examples of the autoradiograms utilized for binding measurements are shown in Figure 2. The tabulation of the causes of death in the autopsy controls has been published (47, 48). Using the databases described above (Fig. 1) and a statistical definition of low binding, we examined the prevalence of low 5-HT_1A_ and 5-HT_2A/C_ binding in SIDS and control infants. It should be noted that the autoradiographic methods used here and previously cannot resolve the binding affinity or levels of receptors on specific cellular phenotypes within the nuclei examined.

### Neuroanatomy

In both Parts I and II, we focused on nuclei in the previously defined medullary 5-HT network comprising the 5-HT source neurons and their medullary target sites (47). The 5-HT system has rostral and caudal domains (47). The rostral domain includes raphe nuclei in the midbrain and pons. Serotonergic neurons in this domain modulate various aspects of cognitive function, sleep, CO_2_ chemosensitivity, arousal, and some aspects of upper airway muscle control. Although this rostral domain plays an important role in cognitive aspects of arousal and consciousness (49), we did not pursue investigation of the rostral domain as our earliest studies did not show consistent binding defects in this region (26). The caudal domain, the focus of our binding studies over the last several decades, contains 5-HT source neurons in the medulla (ROb, raphe magnus [RMg], and raphe pallidus [RPa]), as well as the extra-raphe 5-HT source nuclei in the ventromedial medulla (GC) and ventrolateral medulla (PGCL and intermediate reticular zone [IRZ]), that is reticular formation of the rostral medulla (see Figs. 1 and 2 in Part I (47)). As ROb and RMg have overlapping boundaries, we considered them as a single source nucleus and referred to them as the ROb/RMg in our analyses. This caudal domain is critical for modulation of many physiological functions required for life support, including subcortical arousal which helps support cardiorespiratory homeostasis during sleep periods (14, 50).

### Statistical analysis of low receptor binding

In Part I and earlier publications, we asked whether mean receptor binding in each nucleus differed between infants who died of SIDS and control infants who died of other causes (47). This approach tested for population-level differences between the SIDS and control cohorts but did not assess for abnormalities in individual SIDS infants. Therefore, the current analysis aimed to define “low” on a per-infant and per-nucleus basis, in direct comparison to receptor binding defined as normal in control infants.

We defined low binding as falling below the 95th percentile confidence interval (CI) observed in control tissues. More specifically, because 5-HT_2A/C_ binding in control infants varies with age (47), the CI used was that from regression modeling of binding on postconceptional age, separately for each nucleus. One control infant had excessively high 5-HT_2A/C_ binding (i.e. an outlier) in 6 nuclei and was, therefore, excluded from the modeling. Modeling for 5-HT_1A_ used a quadratic effect of age for greater precision, whereas modeling for 5-HT_2A/C_ used a linear effect, due to the sparseness of the data for the latter. Because laboratory measurements were done in batches over time for each dataset used in prior publications, regression modeling also controlled for dataset. This analysis resulted in an age- and dataset-varying 95% CI around the normal binding levels within control infants. This statistical definition of “normal” is used for a wide variety of laboratory values, but it has, as a consequence, the outcome that some normal (control) babies are expected to have low-binding values by definition (51). Therefore, the study question became, “is the prevalence of low receptor binding greater in infants who died of SIDS compared to infants who died of other causes?”

We defined overall prevalence of low binding in SIDS and control infants based on the percentage of SIDS and control infants that had one or more nuclei statistically defined as having low binding (as defined above). 5-HT_1A_-binding levels were measured in 10 nuclei. While the arcuate nucleus was included in the 10 nuclei measured for 5-HT_1A_, it was not measured for the 5-HT_2A/C_ analysis and thus is not included in the figures. 5-HT_2A/C_-binding levels were measured in 11 nuclei, including 2 different levels of the principal inferior olive ([PIO]; rostral and mid medulla). Only the rostral level of the PIO is included in the figures. We defined the “combined database” as infants with measurements of both 5-HT_1A_ and 5-HT_2A/C_ in the 9 nuclei measured for both (Fig. 1). The prevalence of SIDS and control infants with any binding defined as low was calculated separately for 5-HT_1A_ and 5-HT_2A/C_ and compared between groups via the Fisher exact test. The same data were calculated for the “combined database,” and we further considered whether infants had at least one low binding of either type (5-HT_1A_ or 5-HT_2A/C_). Finally, we considered whether infants had at least one nucleus with low 5-HT_1A_ binding and low 5-HT_2A/C_ binding.

We determined the percentage of nuclei with low binding in each SIDS and control infant. Descriptive statistics of this percentage were calculated separately for 5-HT_1A_ and 5-HT_2A/C_ and compared between groups via t-test. In the combined database, we further considered the percentage of nuclei with low binding for one receptor (5-HT_1A_ or 5-HT_2A/C_) and for both receptors (5-HT_1A_ and 5-HT_2A/C_).

To examine age effects, we defined 3 age groups: early infancy (38–44.9 postconceptional weeks; “Early”), mid infancy (45–59.9 postconceptional weeks; “Mid”), and late infancy (60–76 postconceptional weeks; “Late”). The age groups were chosen based on breaks observed in the 5-HT_2A/C_ age distribution, and on the peak age for SIDS. For example, for an infant born at 37 weeks (term), the mid-infancy group corresponds to 8–22.9 postnatal weeks, roughly the peak age for SIDS.

Postconceptional age was defined as time since (estimated) conception, that is the sum of gestational age (time before birth) and postnatal age (time since birth). Differences between age groups with respect to the percentage of nuclei defined as low binding were assessed via ANOVA, and differences in prevalence of low binding by subnetwork and postconceptional age were tested via logistic regression.

We analyzed 5-HT_1A_ and 5-HT_2A/C_ binding in SIDS infants and controls within 2 serotonergic subnetworks vital for blood pressure regulation: the nucleus of the solitary tract (NTS)-medial accessory olive (MAO)-GC subnetwork and the PGCL-GC-IRZ-ROb source subnetwork. Abnormalities in each circuit were defined in 2 ways: (1) Any nuclei low: scored if at least one nucleus within the subnetwork had low binding, even if data were missing in one or more nuclei; (2) All nuclei low: scored if all nuclei within the subnetwork had low binding; not scored if data were missing in any nuclei. For each subnetwork, significant differences between SIDS and control infants were assessed with Fisher exact tests. The small number of controls prevented us from analyzing the effect of postconceptional age on receptor binding within the subnetworks in controls, but the effect in SIDS infants was tested via logistical regression.

Last, we asked whether risk factors for SIDS (prematurity, sex, prenatal exposure, history of illness within a week before death, prone sleep position, found face down, adult bed/co-sleeping) were associated with particular defects in receptor binding within subnetworks. This analysis was negative (we were unable to define associations between risk factors and specific patterns of receptor binding), and the data are, therefore, not presented or discussed further.

## RESULTS

### 5-HT receptor binding in the medulla in SIDS versus control infants

The results of the 5-HT_2A/C_ receptor binding studies using tissue autoradiography are presented in detail in Part I (47); those data form the basis for the 5-HT_2A/C_ analyses presented here in Part II. Results of the 5-HT_1A_ receptor binding studies are published (28, 48, 52); those data form the basis for the 5-HT_1A_ analyses presented here.

**Figure 3.**
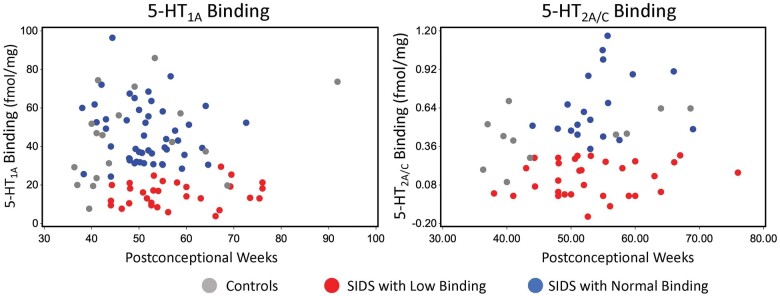
Examples of binding data plotted versus postconceptional age for ^I25^I-DOI binding to 5-HT_2A/C_ receptors and ^3^H-DPAT binding to 5-HT_1A_ receptors in the raphe obscures (ROb/RMg). Controls are shown as gray circles, SIDS infants who statistically have normal binding are shown as blue circles, and SIDS infants who have low binding are shown as red circles. Because the definition of low binding adjusted for laboratory dataset (see Statistical analysis of low receptor binding section), the plots show binding adjusted by dataset for ease of visualization.

Examples of the distribution of receptor binding within the ROb/RMg are shown in Figure 3. 5-HT_1A_ and 5-HT_2A/C_ receptor binding are shown for control infants (gray circles), SIDS infants classified as “normal binding” (blue circles), and SIDS infants classified as “low binding” (red circles). Per the statistical definition of low binding, a fine line exists between normal binding (blue circles) and low binding (red circles) in SIDS infants. There is also overlap between SIDS infants and controls (gray circles).

### Increased prevalence of low 5-HT_1A_ and 5-HT_2A/C_ receptor binding in SIDS infants

The overall prevalence (percentage) of SIDS infants with at least one nucleus defined as having low binding, was ≥77% across all 3 databases (Table 1). In the 5-HT_1A_ database, 79% of SIDS infants have low binding in one or more nuclei, compared to 33% of the control infants (p < 0.001). In the 5-HT_2A/C_ database, 88% of SIDS infants have low binding in one or more nuclei, compared to 42% of control infants (p = 0.001). In the combined database utilizing SIDS infants with both 5-HT_1A_ and 5-HT_2A/C_ receptor binding data available, 87% (45/52) of the SIDS infants had low 5-HT_1A_ receptor binding, 87% (45/52) had low 5-HT_2A/C_ binding, 96% (50/52) had either low 5-HT_1A_ or 5-HT_2A/C_ binding, and 77% (40/52) had both low 5-HT_1A_ and 5-HT_2A/C_ binding. While some control infants had low receptor binding in at least one nucleus, the prevalence of low 5-HT_1A_ and/or 5-HT_2A/C_ binding in the SIDS infants was significantly greater than in the control infants for all 3 databases (p = 0.042 to <0.001, Table 1), and few controls (17%) were identified as having both low 5-HT_1A_ and 5-HT_2A/C_ binding.

**Table 1.** Prevalence of low binding in SIDS versus controls: percent (%) of cases with one or more nuclei defined as low binding

	SIDS		Controls		
	N	%	N	%	**p value** *
Individual databases^†^					
5-HT_1A_	84	79	18	33	<0.001
5-HT_2A/C_	57	88	12	42	0.001
Combined database—cases with both 5-HT_1A_ and 5-HT_2A/C_ data^‡^					
5-HT_1A_	52	87	12	50	0.011
5-HT_2A_	52	87	12	42	0.002
5-HT_1A_ or 5-HT_2A/C_	52	96	12	75	0.042
5-HT_1A_ and 5-HT_2A/C_	52	77	12	17	<0.001

* Fisher exact test.

† The percent of nuclei was calculated based on all available nuclei measured.

‡ The percent of nuclei was calculated based on measured nuclei common to both 5-HT_1A_ and 5-HT_2A/C_.

### SIDS infants have a higher percentage of nuclei with low 5-HT_1A_ and 5-HT_2A/C_ binding

To examine the scope or extent of low receptor binding in infants who died of SIDS compared to infants who died of defined causes, we calculated the number of nuclei per infant with low 5-HT_1A_ or 5-HT_2A/C_ binding. As some infants did not have binding measurements for every nucleus, we calculated the percentage of all nuclei measured that had low binding (Table 2). On average, between 37% and 53% of all nuclei measured in SIDS infants displayed low binding for 5-HT_1A_ or 5-HT_2A/C_ receptors, respectively, in the individual databases. Using the combined database, an average of 79% of nuclei in SIDS infants had either low 5-HT_1A_ or 5-HT_2A/C_ binding and 22% of nuclei had low binding for both 5-HT_1A_ and 5-HT_2A/C_ receptors. By contrast, on average, in the control infants 0%–39% of all nuclei measured had low binding for 5-HT_1A_ and/or 5-HT_2A/C_ receptors in the individual and combined databases. Notably, no nucleus in the control infants had low binding for both 5-HT_1A_ and 5-HT_2A/C_ receptors (Table 2)_._ Overall, SIDS infants had a higher percentage of nuclei with low 5-HT_1A_ and/or 5-HT_2A/C_ binding (p = 0.02 to <0.001) (Table 2).

**Table 2.** Percent (%) of nuclei per infant with low binding in SIDS versus controls

	SIDS					Controls					p value*
	N	Mean	Median	Min	Max	N	Mean	Median	Min	Max	
Individual databases											
5-HT_1A_	84	37	33	0	100	18	12	0	0	70	0.001
5-HT_2A/C_	57	53	64	0	100	12	17	0	0	100	0.002
Combined database—SIDS cases with both 5-HT_1A_ and 5-HT_2A/C_ data											
5-HT_1A_	52	43	39	0	100	12	20	6	0	78	0.020
5-HT_2A_	52	57	71	0	100	12	17	0	0	100	0.002
5-HT_1A_ or 5-HT_2A/C_	52	79	100	0	100	12	39	23	0	100	<0.001
5-HT_1A_ and 5-HT_2A/C_	52	22	13	0	100	12	0	0	0	0	<0.001

* t-Test.

### Overall percentage and prevalence of nuclei with low 5-HT_1A_ and 5-HT_2A/C_ receptor binding

**Figure 4.**
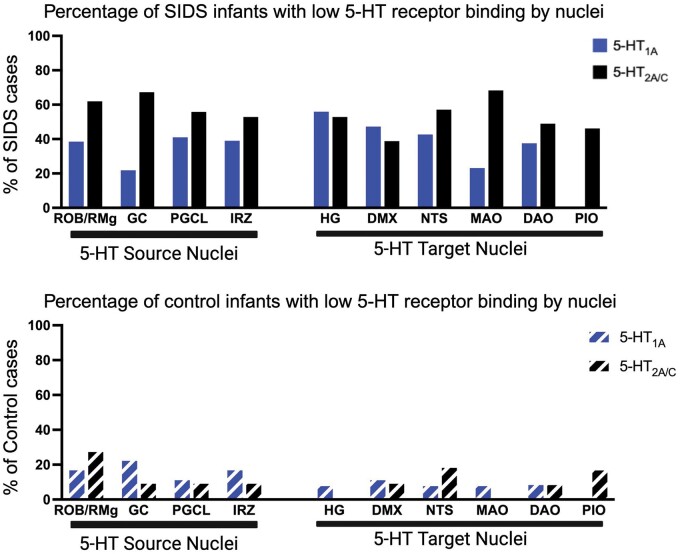
Graphs of percentages of SIDS infants with low-binding values for 5-HT_1A_ and 5-HT_2A/C_ by nuclei in SIDS (top) compared to controls (bottom). Nuclei are separated by “source nuclei” (nuclei containing 5-HT cell bodies) and “target nuclei” (nuclei containing 5-HT terminals without 5-HT cell bodies). The PIO was not measured for 5-HT_1A_. RO, raphe obscurus; GC, nucleus gigantocellularis; PGCL, nucleus paragigantocellularis lateralis; IRZ, intermediate reticular zone; HG, hypoglossal nucleus; DMX, dorsal motor nucleus of vagus; NTS, nucleus of the solitary tract; MAO, medial accessory olive; DAO, dorsal accessory olive; PIO, principal inferior olive. Created with Biorender.com.

Given the distinct functions of specific nuclei in chemoreception, arousal, and/or autoresuscitation, we examined the prevalence of low 5-HT_1A_ and 5-HT_2A/C_ binding across nuclei to determine whether unique patterns existed in SIDS infants compared to controls. To maximize the available data, we utilized the full individual database of SIDS infants from the published 5-HT_1A_ studies (84 SIDS; 18 controls) and the full individual database of SIDS infants from the published 5-HT_2A/C_ study (57 SIDS; 12 controls [Fig. 4]). The percentage of SIDS infants with low 5-HT_1A_ or 5-HT_2A/C_ binding in individual nuclei presented differently across 5-HT source and target nuclei. Within individual nuclei, 22%–56% of SIDS infants had low 5-HT_1A_ receptor binding: the highest prevalence of a 5-HT_1A_ deficit occurred in the hypoglossal nucleus (HG), a 5-HT target nucleus (56%), and the lowest prevalence occurred in the GC, a 5-HT source nucleus (22%). Within individual nuclei, 39%–68% of SIDS infants had low 5-HT_2A/C_ binding: low binding most often appeared in the MAO (68%), followed by GC (67%), ROb/RMg (62%), and the NTS (57%). The least affected nucleus with the lowest prevalence of low 5-HT_2A/C_ binding was the dorsal motor nucleus of vagus (DMX) (39%). Compared to SIDS infants, the percentage of controls with low 5-HT_1A_ or 5-HT_2A/C_ binding in individual nuclei was decreased. Low 5-HT_1A_ binding was identified in 8%–22% of control infants across different nuclei: the GC was most often affected (22%). Similarly, low 5-HT_2A/C_ binding was identified in 0%–27% of control infants across different nuclei, and it occurred most often in ROb/RMg (27%) (Fig. 4).

### Age distribution of the percentage and prevalence of nuclei with low 5-HT_1A_ and 5-HT_2A/C_ receptor binding

**Figure 5.**
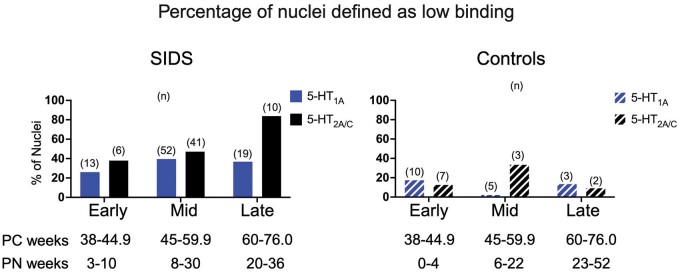
Graphs of percentages of nuclei defined as low binding in SIDS (left) compared to controls (right) for 5-HT_1A_ and 5-HT_2A/C_. The infants are separated into bins based on postconceptional (PC) age; postnatal (PN) age ranges are given for reference. The numbers in each group are indicated above the bar. ROb/RMg, raphe obscurus; GC, nucleus gigantocellularis; PGCL, nucleus paragigantocellularis lateralis; IRZ, intermediate reticular zone; HG, hypoglossal nucleus; DMX, dorsal motor nucleus of vagus; NTS, nucleus of the solitary tract; MAO, medial accessory olive; DAO, dorsal accessory olive; PIO, principal inferior olive. Created with Biorender.com.

Figure 5 shows the percentage of nuclei with low 5-HT_1A_ or 5-HT_2A/C_ binding in SIDS and control infants within 3 different age bins (Early, Mid, and Late). While the percentage of nuclei with low 5-HT_1A_ binding did not vary significantly across age bins (p = 0.35), the percentage of nuclei with low 5-HT_2A/C_ binding was significantly greater in the older infants (“Late”) compared to the younger age bins (“Early” and “Mid”) (p < 0.006) (Fig. 5). The percentage of nuclei with low 5-HT_1A_ and/or 5-HT_2A/C_ binding was less (0%–33%) in control infants across all 3 age bins.

**Figure 6.**
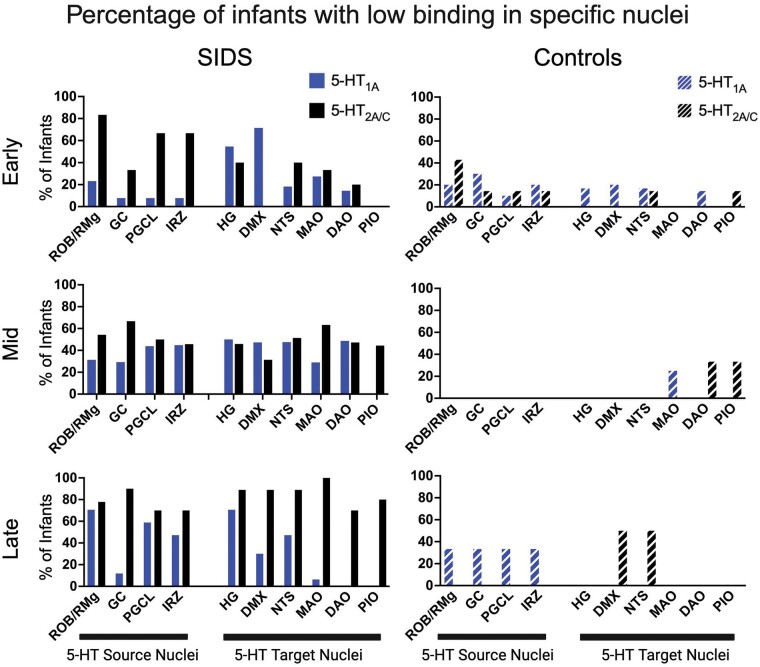
The percentages of infants with low binding for 5-HT_1A_ and 5-HT_2A/C_ in individual nuclei are shown in SIDS (left) compared to controls (right) and divided by age bins (Early, Mid, and Late). Nuclei are separated by “source nuclei” (nuclei containing 5-HT cell bodies) and “target nuclei” (nuclei containing 5-HT terminals without 5-HT cell bodies). The PIO was not measured for 5-HT_1A_. ROb/RMg, raphe obscurus; GC, nucleus gigantocellularis; PGCL, nucleus paragigantocellularis lateralis; IRZ, intermediate reticular zone; HG, hypoglossal nucleus; DMX, dorsal motor nucleus of vagus; NTS, nucleus of the solitary tract; MAO, medial accessory olive; DAO, dorsal accessory olive; PIO, principal inferior olive. Created with Biorender.com.

Figure 6 shows the prevalence (percentage) of infants that showed low binding in specific nuclei across the 3 different age bins. In the youngest SIDS infants (“Early”), low 5-HT_1A_ binding appeared infrequently in the 5-HT source nuclei (ROb/RMg, GC, PGCL, IRZ) but frequently in the 2 target nuclei: DMX (71% of SIDS infants) and HG (55% of SIDS infants). In contrast, low 5-HT_2A/C_ binding occurred much more frequently in the source nuclei of the youngest SIDS infants, especially in the ROb/RMg (67%), PGCL (50%), and IRZ (50%), and less frequently in the DMX (0%) and HG (40%) (Fig. 6). In the oldest SIDS infants (“Late”), the percentage of SIDS with low 5-HT_1A_ binding varied across nuclei (0%–71%) (Fig. 6). For 5-HT_2A/C_ binding, however, the percentage of SIDS cases with low binding was >70% across all nuclei measured. Most notable was the percentage of older SIDS cases with low 5-HT_2A/C_ binding in 5-HT target nuclei: HG (89%), DMX (89%), NTS (89%), and all olivocerebellar nuclei (MAO, PIO, dorsal accessory olive [DAO]) (70%–100% of SIDS infants for each). One hundred percent of the oldest SIDS infants had low 5-HT_2A/C_ binding in the MAO (Fig. 6). In the age bin consistent with the peak period of SIDS (“Mid”), all nuclei displayed some level of vulnerability with prevalence of low binding for either 5-HT_1A_ or 5-HT_2A/C_ being between 29% and 63% in SIDS infants. The prevalence (percentage) of control infants with low binding in specific nuclei, varied with receptor, nuclei, and across age bins. Within younger controls (“Early”), the percentage of controls infants with low binding in either receptor was <30% except for the 5-HT_2A/C_ binding in the ROb/RMg (43%). Low binding was infrequently observed in control infants at the “Mid” and “Late” age bins. An exception includes 5-HT source nuclei in the older infants (ROb/RMg, GC, PGCL, IRZ, all 33.3% for 5-HT_1A_) and the target nuclei in the older infants (DMX and NTS, both 50% for 5-HT_2A/C_) (Fig. 6).

### Prevalence of low binding in specific serotonergic subnetworks

In both SIDS infants and controls, we examined the prevalence of low 5-HT_2A/C_ and 5-HT_1A_ binding within key subnetworks vital for blood pressure regulation (NTS-MAO-GC; that is “olivocerebellar” subnetwork) as well as the subnetwork containing the 5-HT source neurons (ROb/RMg-GC-PGCL-IRZ subnetwork) (53). For each subnetwork, we calculated the percentage of SIDS infants and controls who displayed low receptor binding in any or all nuclei of the subnetwork.

#### Olivocerebellar subnetwork

Table 3 shows the percentage of SIDS and control infants displaying low 5-HT_2A/C_ and 5-HT_1A_ binding in SIDS within each subnetwork. Fifty-one percent of SIDS infants had at least one nucleus in the olivocerebellar subnetwork (NTS-MAO-GC) displaying low 5-HT_1A_ binding, compared to 23% of control infants (p = 0.13). Three percent of SIDS infants displayed low 5-HT_1A_ binding in all component nuclei, similar to control infants (0%; p = 1.0). In contrast, SIDS infants were distinct from controls with respect to 5-HT_2A/C_ binding within the subnetwork: 81% of SIDS infants had at least one of the component nuclei within the olivocerebellar subnetwork with low 5-HT_2A/C_ binding, compared to 18% of control infants (p < 0.001). Most striking, nearly half (46%) of SIDS infants had low 5-HT_2A/C_ binding in all 3 component nuclei of this subnetwork, while there were no controls that displayed low 5-HT_2A/C_ binding in all nuclei (p = 0.008).

**Table 3.** Prevalence of low binding by subnetwork in SIDS versus controls: percent (%) of cases with any or all nuclei defined as low

	**N**	% of SIDS	**N**	% of controls	**p value** ^†^
**NTS-MAO-GC**					
Any nuclei low*					
5-HT_1A_	84	51	18	23	0.12
5-HT_2A/C_	57	81	11	18	<0.001
All nuclei low^‡^					
5-HT_1A_	59	3	13	0	1.00
5-HT_2A/C_	37	46	10	0	0.008
**ROb-GC-PGCL-IRZ**					
Any nuclei low*					
5-HT_1A_	78	53	18	28	0.07
5-HT_2A/C_	52	77	11	27	0.003
All nuclei low^‡^					
5-HT_1A_	77	14	18	6	0.45
5-HT_2A/C_	49	47	11	9	0.038

* Subject was not included if all nuclei had missing data.

‡ Subject was not included if any nuclei had missing data.

† Fisher exact test.

When examined within the previously defined age bins (Early, Mid, and Late), 88% of the oldest SIDS infants (Late) had low 5-HT_2A/C_ binding in all component nuclei of the olivocerebellar circuit compared to 33%–35% of the younger SIDS infants (Early and Mid) (age effect: p = 0.04; Table 4). In comparison, 0%–6% of SIDS infants had low 5-HT_1A_ binding in all of the component nuclei of the network across the Early, Mid, and Late age bins. These analyses demonstrate that low 5-HT_2A/C_ receptor binding is the more common finding in the olivocerebellar subnetwork, especially in the older SIDS cohort.

**Table 4.** Prevalence of low binding by subnetwork and by age: percent (%) of SIDS cases with any or all nuclei defined as low

	**Early** **38–44.9 PC weeks**		**Mid** **45–59.9 PC weeks**		**Late** **60–76 PC weeks**		
	**N** *	% of SIDS	**N** *	% of SIDS	**N** *	% of SIDS	p value^†^
NTS-MAO-GC—any nuclei defined as low							
5-HT_1A_	13	31	52	58	19	47	0.69
5-HT_2A/C_	6	50	41	80	10	100	0.08
NTS-MAO-GC—all nuclei defined as low							
5-HT_1A_	11	0	34	6	14	0	NA
5-HT_2A/C_	3	33	26	35	8	88	0.04
ROb-GC-PGCL-IRZ—any nuclei defined as low							
5-HT_1A_	13	23	48	54	17	71	0.006
5-HT_2A/C_	6	83	36	72	10	90	0.94
ROb-GC-PGCL-IRZ—all nuclei defined as low							
5-HT_1A_	13	8	47	17	17	12	0.82
5-HT_2A/C_	6	33	34	44	9	67	0.63

NA, no test due to inadequate sample size; PC, postconceptional; wks, weeks.

* For the analysis of “any nuclei defined as low,” the subject was not included if data from all nuclei were missing. For the analysis of “all nuclei defined as low,” the subject was not included if data from any nuclei were missing.

† p Value for the effect of PC age, logistic regression.

#### 5-HT source subnetwork

We previously reported that 5-HT_1A_ receptor binding was heavily concentrated in the rostral reticular formation, including the IRZ, PGCL, and GC (source nuclei) compared to the caudal reticular formation (28, 47, 52) (Fig. 2). In the source subnetwork containing the ROb/RMg, GC, PGCL, and IRZ of SIDS infants, the prevalence of low 5-HT_1A_ binding in any nucleus (53%) was not statistically different from its prevalence in controls (28%; p = 0.07; Table 3). Fourteen percent of SIDS infants had low 5-HT_1A_ binding in all component nuclei, which was also no different than the prevalence of low binding in controls (6%; p = 0.45; Table 3). As in the olivocerebellar network, low 5-HT_2A/C_ binding was much more prevalent within the source subnetwork of SIDS infants compared to controls: 77% of SIDS infants displayed low 5-HT_2A/C_ binding in any of the nuclei, compared to 27% of controls (p = 0.003). Similar to the olivocerebellar subnetwork, nearly half (47%) of SIDS infants had low HT_2A/C_ binding in all component nuclei of the source subnetwork, compared to 9% of controls (p = 0.038; Table 3).

Seventy-one percent of the oldest SIDS infants had low 5-HT_1A_ binding in any nuclei of this source subnetwork, compared to 23% and 54% of the younger SIDS infants in the Early and Mid bins, respectively (age effect: p = 0.006) (Table 4). There was no difference in prevalence of low 5-HT_2A/C_ receptor binding across age bins in the source subnetwork. These analyses demonstrate that low 5-HT_2A/C_ and 5-HT_1A_ binding occur frequently in the 5-HT source subnetwork; the prevalence of low 5-HT_1A_ binding emerges to a greater extent in the older SIDS cohort.

## DISCUSSION

In Part I of this series, we reported that SIDS infants have altered 5-HT_2A/C_ binding in key medullary nuclei supporting chemoreception, arousal, and autoresuscitation, in addition to altered 5-HT_1A_ binding reported previously (47). In Part II, we address the hypothesis that low 5-HT_1A_ and 5-HT_2A/C_ binding manifest differentially in serotonergic source and target nuclei of SIDS infants across the 3 age bins examined. For each nucleus, we defined the low binding as binding below the lower boundary of the 95% CI of the control infant binding. Utilizing previously published data in Part I and new statistical approaches, we provide evidence that: (1) compared to control infants, SIDS infants have a greater prevalence of low 5-HT_1A_ or 5-HT_2A/C_ binding; (2) the percentage of nuclei with low 5-HT_1A_ or 5-HT_2A/C_ binding was 2–3 times greater in SIDS infants compared to controls; and (3) 5-HT source nuclei exhibited a higher prevalence of low 5-HT_2A/C_ binding compared to 5-HT_1A_. Related to postconceptional age, we showed: (1) the low 5-HT_1A_ binding was observed more frequently in the HG and DMX of the youngest SIDS infants but, with the exception of the GC, was more prevalent in the source nuclei of the oldest SIDS infants; (2) in the source nuclei of the youngest SIDS infants, low 5-HT_2A/C_ binding occurred much more frequently than low 5-HT_1A_ binding; (3) in the oldest SIDS infants, low 5-HT_2A/C_ binding was more prevalent across all source and target nuclei, with low 5-HT_2A/C_ binding in the MAO notably observed in 100% of older SIDS infants; (4) that at ages consistent with the peak risk of SIDS (Mid), the prevalence of low binding was widespread (∼30%–70% of SIDS infants) through all measured nuclei for both 5-HT_1A_ and 5-HT_2A/C_ receptors; and (5) nearly half of all SIDS infants had low 5-HT_2A/C_ binding in every component nucleus of the source (ROb/RMg-GC-PGCL-IRZ) and olivocerebellar subnetworks (NTS-MAO-GC). These findings support the hypothesis that the patterns of low 5-HT_1A_ and 5-HT_2A/C_ binding are distinct, appearing differentially in 5-HT source and target nuclei and differentially as a function of age at the time of death.

### 5-HT_1A_ and 5-HT_2A/C_ receptors: balancing excitation and inhibition in neural circuits involved in chemoreception, arousal, and autoresuscitation

5-HT_1A_ and 5-HT_2A/C_ receptors have been a focus of our research because they are expressed in key medullary nuclei and participate in the maintenance of cardiorespiratory homeostasis in sleep, including processes involved in chemoreception, arousal, and autoresuscitation. 5-HT_2A/C_ receptors are excitatory, activating downstream signaling pathways that are permissive for several excitatory post-synaptic currents, including glutamate and persistent sodium currents (14). In this way, 5-HT_2A/C_ receptor activation facilitates the bursting of pacemaker and other neurons in the Pre-Bötzinger complex (PreBotC) (45, 54, 55), presympathetic neurons (56, 57), and specific neurons that promote arousal (9). On the other hand, 5-HT_1A_ receptors are generally inhibitory and, unlike 5-HT_2A/C_ receptors, are expressed somato-dendritically as autoreceptors, reducing the activity of 5-HT neurons. 5-HT_1A_ receptors are also expressed by inhibitory GABAergic and glycinergic neurons at target nuclei, and their activation can dampen inhibitory neurotransmission at these sites (58, 59). These concepts should be considered with respect to the functional consequences of reduced 5-HT_1A_ binding in SIDS infants within nuclei critical for arousal, cardiorespiratory homeostasis, and autoresuscitation (described in the next section). For example, reduced 5-HT_1A_ activity may augment inhibitory neurotransmission at target sites, compromising the function of these critical processes. It is also possible that, as an autoreceptor, reduced 5-HT_1A_ activity in the source nuclei could increase 5-HT release at target sites.

### Consequences of reduced 5-HT_1A_ and 5-HT_2A/C_ binding on chemoreception, arousal, and autoresuscitation

Pathological findings coupled with analyses of cardiorespiratory records obtained from SIDS infants immediately prior to death suggest that at least a subset of SIDS infants die from severe hypoxemia that is not reversed by gasping. Gasping occurs, but there is a failure of at least one of the cardiovascular or autonomic processes that support and are necessary for autoresuscitation and survival (24, 25). The data presented here in Part II are in keeping with previous findings from our group suggesting that serotonergic dysfunction—that is reduced drive from 5-HT source neurons and compromised 5-HT_1A_ and 5-HT_2A/C_ signaling in the 5-HT target nuclei—is highly associated with a substantial proportion of SIDS deaths. It is worth noting from previous studies that SIDS is not associated with a loss of receptor binding globally; for example, both alpha2 adrenergic (60) and µ-opioid receptor binding (61) are normal in SIDS infants.

5-HT neurons in the reticular formation of the rostral ventral medulla (i.e. ROb/RMg, IRZ, GC, and PGCL) innervate the nuclei in which we assessed 5-HT_1A_ and 5-HT_2A/C_ binding. Serotonergic source nuclei and subnetworks (i.e. circuits) that they innervate bestow the infant with an array of physiological responses that improve brain oxygenation, including arousal, O_2_, and CO_2_ chemoreflex-induced increases in ventilation and apnea termination, gasping, sympathoexcitation, regulation of heart rate, and contractility and cerebral vasodilation. Reduced 5-HT_1A_ or 5-HT_2A/C_ activity in target nuclei of critical subnetworks may compromise these processes. Alternatively, reduced serotonergic drive to the targets, due to either reduced 5-HT and/or immature 5-HT neurons, may be the primary defect, with altered receptor expression representing a secondary response. With this caveat in mind, below we suggest potential physiological consequences of low 5-HT_1A_ and 5-HT_2A/C_ binding for an infant during sleep.

#### Low receptor binding in key motor nuclei

In the current study, the patterns of low 5-HT_1A_ and 5-HT_2A/C_ binding are distinct in different 5-HT source and target nuclei and depend on age. In the youngest SIDS infants, low 5-HT_2A/C_ binding appears frequently in 5-HT source nuclei, with low 5-HT_1A_ binding appearing most often in 2 major motor nuclei: the HG which provides drive to the tongue and a number of upper airway muscles to reduce airway resistance during inspiration, and the DMX which contains neurons that provide parasympathetic control of the viscera. As described in the previous section, 5-HT_1A_ receptor activation leads to neuronal inhibition, whether on 5-HT neurons themselves or neurons within target nuclei, some of which may be inhibitory. Glycinergic and GABAergic neurons provide tonic inhibitory drive to HG neurons in sleep (62, 63). Inhibition from 5-HT_1A_ receptors may constrain GABAergic and glycinergic inhibitory activity within the HG of infants; reduced 5-HT_1A_ activity on these inhibitory neurons may augment GABAergic or glycinergic tone, compromising airway patency. This scenario is plausible when considering previous studies on the role of 5-HT_1A_ in respiratory circuits, including HG neurons (59, 64), but requires evaluation in animal models at ages relevant to SIDS.

The prevalence of low 5-HT receptor binding was more widespread across source and target nuclei at mid-infancy (when the risk of SIDS increases) and in the oldest cohort of SIDS infants, particularly for 5-HT_2A/C_. One potential consequence of this is total network insufficiency during severe apnea and bradycardia, rendering the infant incapable of reoxygenating the brain.

### Low receptor binding in the NTS and olivocerebellar subnetwork

**Figure 7.**
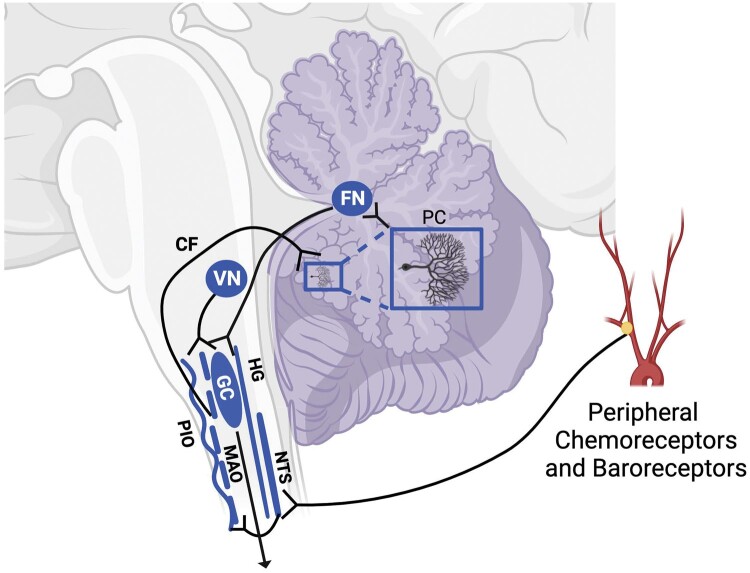
Schematic diagram of vestibular nucleus (VN), nucleus of solitary tract (NTS), medial accessory olive (MAO), climbing fiber (CF), Purkinje cells (PC), fastigial nuclei (FN), and gigantocellularis (GC) circuitry that underlie dampening and recovery for blood pressure changes signaled by the NTS and VN. Change in body position (e.g. prone versus supine), or factors inducing shock are mediated by the VN and NTS. Signals are transmitted to MAO neurons and then to Purkinje cells, via climbing fibers, that project to FN. FN induces changes in autonomic motor output, body movement, arousal and upper airway tone via projections to GC and HG, among others. The circuitry is also sensitive to chemoreceptor activation, with the FN terminating prolonged apnea periods. Created with Biorender.com.

A striking finding was that ∼3/4 of SIDS infants had low 5-HT_2A/C_ binding in at least one component nucleus of the olivocerebellar subnetwork (NTS-MAO-GC), and nearly half of SIDS cases had low 5-HT_2A/C_ binding in all 3 component nuclei. Low 5-HT_2A/C_ binding was far less prevalent in the olivocerebellar subnetwork of control infants: ∼1/4 had low binding in at least one component nucleus, and we could identify no infants who had low binding in all 3. This difference between SIDS and control infants was unique to 5-HT_2A/C_ binding. The NTS is critical for baroreflex-mediated control of arterial blood pressure and 5-HT, acting through 5-HT_2A_ receptors in the NTS, facilitates the baroreflex (65), in part via subsequent neuromodulation at the rostral ventrolateral medulla (RVLM) (65). 5-HT may also act at the NTS to facilitate the sympathoexcitation required to reverse the fall in arterial blood pressure during severely hypoxic conditions (66). Within the olivocerebellar subnetwork (Fig. 7), the NTS acts in conjunction with the MAO and the cerebellar fastigial nucleus (FN) to restore blood pressure in a variety of physiologic and pathophysiological contexts (67, 68), in part through interactions with presympathetic neurons in the RVLM (69). Although our group has not specifically investigated the FN of the cerebellum, 5-HT_2A/C_ receptors are expressed in this nucleus, and their activation is sufficient to alter the activity of FN neurons and modify behavior (70). Thus, reduced drive through 5-HT_2A/C_ receptors within the NTS and/or other components of the olivocerebellar subnetwork may negatively impact multiple, integrated processes that facilitate blood pressure recovery following a hypotensive event, including those events associated with severe hypoxia.

The functional consequences of potential 5-HT_2A/C_ dysfunction within the olivocerebellar subnetwork are especially intriguing when considering the pathological findings at the death scene (71). In addition to the evidence from cardiorespiratory records suggesting cardiovascular collapse, additional signs of autonomic dysfunction have been documented, including a “shock-like” appearance: marked sweating with pallor, indicative of a sympathetic burst followed by blood pressure loss (72). Prone positioning is a clear risk factor for SIDS (73). It is possible that SIDS infants have an impaired vestibular response to such body positioning that would compromise the control of blood pressure in the prone versus supine position, especially if critical components of the vestibular-cerebellar network, for example the MAO, rely on drive through 5-HT_2A/C_ receptors. In normal infants, input from the vestibular system targets the MAO, which subsequently provides drive to Purkinje cells (via climbing fibers) that, in turn, modulate deep cerebellar FN activity to regulate blood pressure and heart rate or terminate apnea (Fig. 7); part of that regulation is to dampen extreme alterations in blood pressure, including recovery from marked hypotension (71).

#### Low receptor binding in 5-HT source nuclei

**Figure 8.**
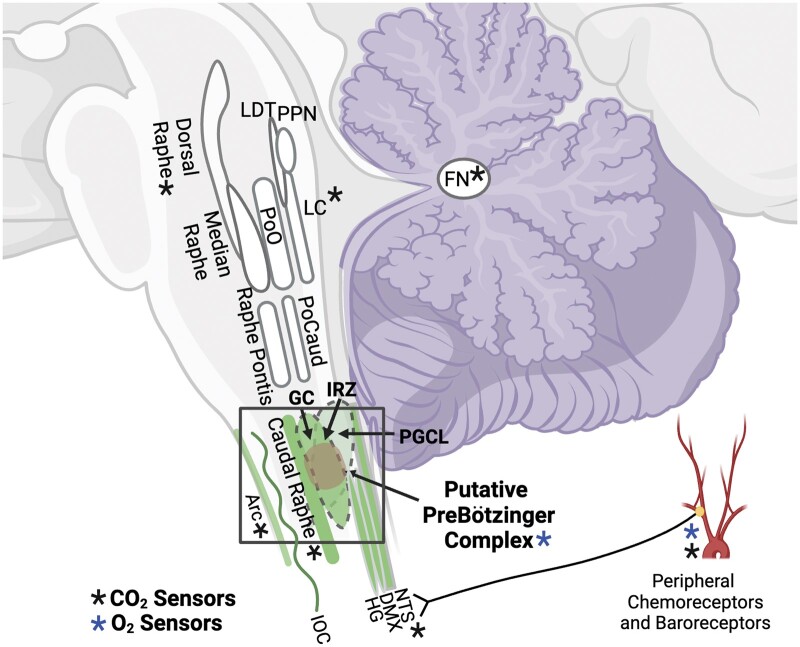
Schematic representation of regional involvement of sites (nuclei) with abnormal 5-HT_1A_ and/or 5-HT_2A/C_ binding in SIDS infants compared to autopsy controls. Notably all of the involved sites are involved in protective responses to asphyxia and modulated by 5-HT in the putative subset of SIDS representing a serotonopathy (see text). The key site in SIDS infants is the mid-to-rostral medulla (rectangle), a critical (“segmental”) feature of the 5-HT-related pathology uncovered by us in SIDS over at least 2 decades of research, including in Parts I and II. The nuclei with abnormal 5-HT receptor binding in the SIDS cases are denoted in green. The RVLM is included in this regional tissue “block” (rectangle) of the hindbrain that is affected in the SIDS and includes the anatomic loci of the putative human homologue of the pre-Bötzinger complex, intermediate reticular zone (IRZ), paragigantocellularis lateralis (PGCL), and more medially, gigantocellularis (GC). The rostral medulla also contains the major 5-HT-synthesizing (source) neurons in the caudal (medullary [as opposed to the rostral (mesopontine dorsal and median raphe)]) domains of the brainstem serotoninergic system, that is caudal raphe, IRZ, PGCL, and GC. The caudal raphe includes the raphe obscurus, raphe magnus, and raphe pallidus. Of note, the source neurons and other affected (non-source) nuclei in SIDS cases (i.e. hypoglossal nucleus [HG], dorsal motor nucleus of the vagus [DMX], nucleus of the solitary tract [NTS], and inferior olivary complex [IOC]), receive 5-HT (target) projections, albeit not exclusively in the entire brainstem or forebrain (not shown). The non-HT-source (target) nuclei of the HG, NTS, DMX, and IOC all demonstrate abnormal 5-HT_1A_ and/or 5-HT_2A/C_ receptor binding in the SIDS cases, anatomic portions of which are included in the affected hindbrain segment (rectangle) of the rostral medulla. The medial accessory olive (MAO), which is a critical component of the affected olivocerebellar circuit involved in blood pressure recovery (see text), is included in the IOC. The major sites involved in chemosensory processing are highlighted with notation in blue asterisk for central and peripheral oxygen (O_2_) (hypoxia) sensors and black asterisk for carbon dioxide (CO_2_) (hypercapnia) sensors. Peripheral chemoreceptors are located in the carotid body, and peripheral baroreceptors in the carotid sinus and aortic arch. Important sites of chemosensory circuitry in the abnormal hindbrain segment in the SIDS cases (rectangle), support their role in the putative defective defense responses in SIDS. The retrotrapezoid nucleus, known to be essential for brainstem CO_2_ chemosensitivity (98) is not shown in this diagram because the anatomic locus of its site in humans is not resolved, and thus receptor autoradiography and other studies have yet to be performed in SIDS versus controls. This schematic representation makes the key points of the results of Parts I and II in SIDS cases that brainstem: (1) serotoninergic-related pathology reported to date by us appears to be concentrated in the mid-to-rostral medulla; (2) is segmentally constricted in the medullary hindbrain; and (3) involves essential anatomic loci that mediate serotoninergic defense responses in arousal, chemosensitivity, autoresuscitation, and cardiopulmonary reflexes, many of which are operative during a sleep period. Other abbreviations include: PoO, pontis oralis; LDT, lateral dorsal tegmentum; PPN, pedunculopontine nucleus; PoC, pontis caudalis; LC, locus coeruleus; FN, fastigial nuclei. Created with Biorender.com.

The relevance of low 5-HT_2A/C_ binding within the IRZ and GC are worth highlighting specifically, as these nuclei likely contain critical groups of neurons required in human infants for the ventilatory response to hypercapnia (32), full arousal, and successful autoresuscitation. Although it has yet to be convincingly identified in the human medulla, by extrapolating the 3-dimensional region containing the preBotC in the rodent medulla, the human preBotC, and presympathetic neurons in the RVLM are likely contained within the IRZ and GC (Fig. 8). In rodents, the preBotC is recognized as a cluster of interneurons within the ventrolateral medulla (i.e. the equivalent of the IRZ in humans) that drives gasping via intrinsically bursting, hypoxia resistant “pacemaker” neurons (74, 75). The preBotC is a target of 5-HT neurons and its output is stimulated by 5-HT projections (44, 50). During autoresuscitation, gasping is required to rapidly increase pulmonary ventilation and the diffusion of O_2_ into the blood. As important as it may be, gasping alone is insufficient for surviving severe hypoxia. Fortuitously, for the normal infant, the preBotC operates in an integrative fashion with the presympathetic neurons in the RVLM to drive phasic increases in sympathetic nerve activity during severe hypoxia (76). The RVLM, in turn, activates neurons in the cerebral vasodilating region of the medulla to ensure sufficient delivery of newly oxygenated arterial blood to the brain (77). 5-HT_2A/C_ activation in the rodent preBotC is permissive to the bursting of hypoxia resistant pacemaker neurons that drive gasping in conscious animals (54, 55) as well as the coupled phasic increase in sympathetic activity. In controls, 5-HT_2A/C_ binding is concentrated in the rostral aspect of the ventral medulla—an area that contains the 5-HT source subnetwork (ROb/RMg-GC-PGCL-IRZ) and as discussed above, likely contains the preBotC (Fig. 2). If low 5-HT_2A/C_ binding in this region reflects low receptor activity in vivo, then gasping and the sympathetic response to severe hypoxia may be compromised, preventing successful autoresuscitation and possibly culminating in SIDS.

The prevalence of low receptor binding across the medullary nuclei differs at the 3 age intervals examined. For example, in the youngest SIDS infants, low 5-HT_1A_ binding was most prevalent in 2 target nuclei (the HG and DMX), while low 5-HT_2A/C_ binding was most prevalent in the 5-HT source nuclei. In contrast, in the eldest SIDS infants low 5-HT_2A/C_ binding was highly prevalent across all target nuclei. Development strongly impacts aspects of cardiorespiratory control in infancy (78). In the youngest infants, death may result with relatively minor or circumscribed receptor defects. As neural networks controlling cardiorespiratory function and arousal develop and become more adult-like, a more extensive loss of serotonergic function may be required to compromise chemoreflexes, arousal, and autoresuscitation that together protect an infant during sleep periods. It is also unknown whether older infants acquired more extensive receptor defects with time or whether they simply did not encounter a specific environmental stressor or combination of stressors that would have precipitated death at a younger age.

### Limitations

Our statistical approach was devised to define the prevalence and extent of statistically defined low 5-HT receptor binding in the medulla of SIDS. However, the limitations of this approach must be noted. Our definition of “low” was based on a small dataset of controls that may not themselves be normal, having succumbed to known and varied causes of death as described in more detail in Part I (47). The CI of control data is large due to the small number of control infants, and some controls were identified as falling below this CI because of a statistical definition of normal based on 95% confidence intervals; our ability to define “low” receptor binding and to differentiate true differences between low and “normal” receptor bindings is modest. Furthermore, the relatively small sample size for the younger and older age groups (compared to the mid-infancy age group) results in low power for testing across age and multiple testing within the small sample. Additionally, the CI used for controls is not an ideal definition of low receptor binding since it is the CI for the expected value of the bindings, not for the binding itself. The lower bound of the prediction interval for the regression of receptor bindings on postconceptional age in controls would be the more appropriate metric; however, given the small sample size, the prediction interval was extremely wide, and this method identified few infants as low. Finally, because laboratory measurements were done in batches over time, regression modeling was used to control for dataset; however, batch effects may not have been constant across all infants.

The data utilized in this study were examined previously for abnormalities of 5-HT receptors in the key medullary regions involved in chemoreception, arousal, and cardiorespiratory function. However, other neurotransmitter systems and nuclei may be abnormal in SIDS compared to controls—these nuclei and neurotransmitters may play a role, direct or indirect, in serotonergic function (79–93). Given the significant risk of nicotine exposure for SIDS (94), and the known neuroanatomical deficiencies in a high-risk population with such exposure (95, 96), interactions between 5-HT and nicotinic acetylcholine receptors are a special concern. Finally, while our work has focused on the medulla, regions rostral to the medulla (i.e. pons and limbic forebrain—hypothalamus, hippocampus, and amygdala), and cerebellum are critical for other processes (e.g. sleep, protective cardiac, and respiratory reflexes) not necessarily involved in autoresuscitation but possibly affected in SIDS (83, 97–100). Despite these limitations, the data provide novel insight into a subset of SIDS infants identified by our previous studies as having a serotonopathy.

### Conclusions and future directions

In Parts I and II of this 2-part series, our aim was to utilize tissue autoradiography from the medulla of SIDS infants to further test the hypothesis that 5-HT_1A_ and 5-HT_2A/C_ receptor binding is altered in SIDS cases as compared to controls, which could underlie a failure to arouse and/or autoresuscitate leading to death. Information regarding such biological abnormalities is essential for the potential development of pharmacological or neuromodulatory interventions that aim to prevent or minimize SIDS in at-risk populations. If we equate our statistically defined low receptor binding with a receptor binding deficit or abnormality, we propose that the key serotonergic receptor deficits that we have identified are important surrogate markers. We further propose that these surrogate markers identify subsets of SIDS infants that are burdened with serotonergic defects within critical subnetworks supporting cardiorespiratory function; in essence, these defects represent a “serotonopathy.” Indeed, serotonopathies with variable origins (i.e. receptor deficits, altered 5-HT release, immature 5-HT neurons among others) may reflect recently proposed “endophenotypes” of pediatric and adult diseases of genetic origin (101). Infants harboring a serotonergic abnormality may survive the critical period of SIDS; they may have avoided a key exogenous stressor that might otherwise expose their latent vulnerability. They may nevertheless remain susceptible to a diverse array of diseases manifesting with unique, age- or developmentally dependent trajectories to the extent those disorders rely on serotonergic activity. We stress that our proposed “serotonopathy” hypothesis of SIDS, while not necessarily of genetic origin, is generally in keeping with this endophenotype concept.

Our overall goal as a group has been to provide novel conceptual insights on the possible pathophysiological mechanisms that lead to SIDS, using neurochemical findings from SIDS infants and controls (e.g. the alterations in 5-HT receptor binding activity described in Part I and herein), coupled with mechanistic experimental data from animal models in which these subnetworks have been perturbed. Indeed, leaning heavily on what we have learned as a group from modeling serotonergic dysfunction in animals (30–35, 37–42, 50, 102–107), we believe that dysfunction within the medullary 5-HT system represents an underlying inherent vulnerability that may intersect with an exogenous stressor at a critical period of infant development, with the outcome manifesting as SIDS (i.e. Triple Risk Model of SIDS (108)). Until novel diagnostic methods are developed to reliably identify serotonergic and other biomarkers of SIDS that help guide biological treatments, behavioral modifications that ensure safe sleep environments (i.e. supine sleep position, no bed-sharing) should continue to be an integral component of the quest for the total eradication of SIDS.
